# Encoding Sequential Information in Semantic Space Models: Comparing Holographic Reduced Representation and Random Permutation

**DOI:** 10.1155/2015/986574

**Published:** 2015-04-07

**Authors:** Gabriel Recchia, Magnus Sahlgren, Pentti Kanerva, Michael N. Jones

**Affiliations:** ^1^University of Cambridge, Cambridge CB2 1TN, UK; ^2^Swedish Institute of Computer Science, 164 29 Kista, Sweden; ^3^Redwood Center for Theoretical Neuroscience, University of California, Berkeley, Berkeley, CA 94720, USA; ^4^Indiana University, Bloomington, IN 47405, USA

## Abstract

Circular convolution and random permutation have each been proposed as neurally plausible binding operators capable of encoding sequential information in semantic memory. We perform several controlled comparisons of circular convolution and random permutation as means of encoding paired associates as well as encoding sequential information. Random permutations outperformed convolution with respect to the number of paired associates that can be reliably stored in a single memory trace. Performance was equal on semantic tasks when using a small corpus, but random permutations were ultimately capable of achieving superior performance due to their higher scalability to large corpora. Finally, “noisy” permutations in which units are mapped to other units arbitrarily (no one-to-one mapping) perform nearly as well as true permutations. These findings increase the neurological plausibility of random permutations and highlight their utility in vector space models of semantics.

## 1. Introduction

Semantic space models (SSMs) have seen considerable recent attention in cognitive science both as automated tools to estimate semantic similarity between words and as psychological models of how humans learn and represent lexical semantics from contextual cooccurrences (for a review, see [1]). In general, these models build abstract semantic representations for words from statistical redundancies observed in a large corpus of text (e.g., [2, 3]). As tools, the models have provided valuable metrics of semantic similarity for stimulus selection and control in behavioral experiments using words, sentences, and larger units of discourse [4–6]. As psychological models, the vectors derived from SSMs serve as useful semantic representations in computational models of word recognition, priming, and higher-order comprehension processes [7–12]. In addition, the semantic abstraction algorithms themselves are often proposed as models of the cognitive mechanisms used by humans to learn word meaning from repeated episodic experience, although there has been criticism that this theoretical claim may be overextending the original intention of SSMs [13–15].

A classic example of an SSM is Landauer and Dumais' [2] latent semantic analysis model (LSA). LSA begins with a word-by-document matrix representation of a text corpus, where a word is represented as a frequency distribution over documents. Next, a lexical association function is applied to dampen the importance of a word proportionate to its entropy across documents (see [16] for a review of functions used in various SSMs). Finally, singular value decomposition is applied to the matrix to reduce its dimensionality. In the reduced representation, a word's meaning is a vector of weights over the 300 latent dimensions with the largest eigenvalues. The dimensional reduction step has the effect of bringing out latent semantic relationships between words. The resulting space positions words proximally if they cooccur more frequently than would be expected by chance and also if they tend to occur in similar semantic contexts (even if they never directly cooccur).

More recent SSMs employ sophisticated learning mechanisms borrowed from probabilistic inference [18], holographic encoding [19], minimum description length [20], random indexing [21], and global memory retrieval [22]. However, all are still based on the fundamental notion that lexical semantics may be induced by observing word cooccurrences across semantic contexts [23, 24], and no single model has yet proven itself to be the dominant methodology [1].

Despite their successes both as tools and as psychological models, current SSMs suffer from several shortcomings. Firstly, the models have been heavily criticized in recent literature because they learn only from linguistic information and are not grounded in perception and action; for a review of this debate, see de Vega et al. [25]. The lack of perceptual grounding is clearly at odds with the current literature in embodied cognition, and it limits the ability of SSMs to account for human behavior on a variety of semantic tasks [26]. While the current paper does not address the issue of incorporating perceptual grounding into computational models trained on linguistic data, the issue is discussed at length in several recent papers (e.g., [15, 27–30]). Secondly, SSMs are often criticized as “bag of words” models because (with the exception of several models to be discussed in the next section) they encode only the contexts in which words cooccur, ignoring statistical information about the temporal order of word use within those contexts. Finally, many SSMs suffer from a difficulty to scale to linguistic data comparable to what humans experience. In this paper, we simultaneously address order and scalability in SSMs.

*The Role of Word Order in Lexical Semantics*. A wealth of evidence has emphasized the importance of domain-general sequential learning abilities in language processing (see [31, 32], for reviews), with recent evidence suggesting that individual differences in statistical sequential learning abilities may even partially account for variations in linguistic performance [33]. Bag of words SSMs are blind to word order information when learning, and this has been criticized as an “architectural failure” of the models [13] insofar as it was clear a priori that humans utilize order information in almost all tasks involving semantic cognition. For example, interpretation of sentence meaning depends on the sequential usage tendencies of the specific component words [34–40].

One common rebuttal to this objection is that order information is unimportant for many tasks involving discourse [2]. However, this seems to apply mostly to applied problems with large discourse units such as automatic essay grading [41]. A second rebuttal is that SSMs are models of how lexical semantics are learned and represented, but not how words are used to build sentence/phrase meaning [42, 43]. Hence, order is not typically thought of as a part of word learning or representation, but rather how lexical representations are put together for comprehension of larger units of discourse. Compositional semantics is beyond the scope of SSMs and instead requires a process account of composition to build meaning from SSM representations, and this is the likely stage at which order plays a role [9, 11].

However, this explanation is now difficult to defend, given a recent flurry of research in psycholinguistics demonstrating that temporal order information is used by humans when learning about words, and that order is a core information component of the lexical representation of the word itself. The role of statistical information about word order was traditionally thought to apply only to the rules of word usage (grammar) rather than the lexical meaning of the word itself. However, temporal information is now taking a more prominent role in the lexical representation of a word's meaning. Elman [44] has recently argued that the lexical representations of individual words contain information about common temporal context, event knowledge, and habits of usage (cf. [4, 45–47]). In addition, recent SSMs that integrate word order information have seen greater success at fitting a human data in semantic tasks than SSMs encoding only contextual information (e.g., [16, 19, 48–50]).

*The Role of Data Scale in Lexical Semantics*. SSMs have also been criticized due to their inability to scale to realistic sizes of linguistic data [51, 52]. The current corpora that SSMs such as LSA are frequently trained on contain approximately the number of tokens that children are estimated to have experienced in their ambient environment by age three (in the range of 10–30 million), not even including words produced by the child during this time [14, 53]. Given that SSMs are typically evaluated using benchmarks elicited from college-age participants, it would be ideal if they were trained upon a quantity of linguistic input approximating the experience of this age.

However, SSMs that rely on computationally complex decomposition techniques to reveal the latent components in a word-by-document matrix (e.g., singular value decomposition) are not able to scale up to corpora of hundreds of millions of tokens, even with high-end supercomputing resources. Although new methods for scaling up singular value decomposition to larger input corpora have shown promise [54, 55], there will always be a practical upper limit to the amount of data that can be processed when compared to continuous vector accumulation techniques. The problem is exacerbated by the fact that as the size of the corpus increases, the numbers of rows and columns in the matrix both increase significantly: the number of columns grows linearly in proportion to the number of documents, and the number of rows grows approximately in proportion to the square root of the number of tokens (Heap's law).

As the availability of text increases, it is an open question whether a better solution to semantic representation is to employ simpler algorithms that are capable of both integrating order information and scaling up to take advantage of large data samples or whether time would better be spent optimizing decomposition techniques. Recchia and Jones [52] demonstrated that although using an extremely simple method (a simplified version of pointwise mutual information) to assess word pairs' semantic similarity was outperformed by more complex models such as LSA on small text corpora, the simple metric ultimately achieved better fits to human data when it was scaled up to an input corpus that was intractable for LSA. Similarly, Bullinaria and Levy [16] found that simple vector space representations achieved high performance on a battery of semantic tasks, with performance increasing monotonically with the size of the input corpus. In addition, Louwerse and Connell's [56] simulations indicated that first-order cooccurrence structure in text was sufficient to account for a variety of behavioral trends that had seemed to be indicative of a “latent” learning mechanism, provided that the text learned from was at a sufficiently large scale. These findings were one factor that led these authors to favor simple and scalable algorithms to more complex nonscalable algorithms.

The issue of scalability is more than simply a practical concern of computing time. Connectionist models of semantic cognition (e.g., [57, 58]) have been criticized because they are trained on “toy” artificial languages that have desirable structure built-in by the theorist. These small training sets do not contain the complex structure inherent in real natural language. To produce humanlike behavior with an impoverished training set, the models are likely to be positing overly complex learning mechanisms compared to humans who learn from experience with much larger amounts of complex linguistic data. Hence, humans may be using considerably simpler learning mechanisms because much of the requisite complexity to produce their semantic structure is the result of large sampling from a more complex dataset [14, 19]. A model of human learning should be able to learn data at a comparable scale to what humans experience, or it risks being overly complex. As Onnis and Christiansen [59] have noted, many models of semantic learning “assume a computational complexity and linguistic knowledge likely to be beyond the abilities of developing young children” [59, abstract].

The same complexity criticism applies to most current SSMs. Although they learn from real-world linguistic data rather than artificial languages, the amount of data they learn from is only about 5% of what is likely experienced by the college-age participants who produce the semantic data that the models are fit to. Of course, a strict version of this argument assumes equal and unchanging attention to incoming tokens, which is unlikely to be true (see [7, 60]). Hence, to produce a good fit to the human data with impoverished input, we may be developing SSMs that have unnecessary complexity built into them. This suggestion explains why recent research with* simple and scalable* semantic models has found that simple models that scale to large amounts of data consistently outperform computationally complex models that have difficulty scaling (e.g., [51, 52]; cf. [61]).

## 2. Methods of Integrating Word Order into SSMs

Early work with recurrent neural networks [57, 62–64] demonstrated that paradigmatic similarity between words could be learned across a distributed representation by attending to the sequential surroundings of the word in the linguistic stream. However, this work was limited to small artificial languages and did not scale to natural language corpora. More recently, work by Howard and colleagues with temporal context models [65–68] has shown promise at applying neurally inspired recurrent networks of temporal prediction by the hippocampal system to large real-world language corpora. Tong and colleagues have demonstrated the utility of echo state networks in learning a grammar with long-distance dependencies [69], although their work focused on a corpus of an artificial language similar to that of Elman [70]. In a similar vein, liquid state machines have been successfully trained upon a corpus of conversations obtained from humans performing cooperative search tasks to recognize phrases unfolding in real time [71].

Other noteworthy works on distributional representations of word meaning include “deep learning” methods [72], which have attracted increasing attention in the artificial intelligence and machine learning literature due to their impressive performance on a wide variety of tasks (see [73, 74] for reviews). Deep learning refers to a constellation of related methods for learning functions composed of multiple nonlinear transformations by making use of “deep” (i.e., highly multilayered) neural networks. Intermediate layers, corresponding to intermediate levels of representation, are trained one at a time with restricted Boltzmann Machines, autoencoders, or other unsupervised learning algorithms [72, 75, 76]. These methods have been applied to construct distributed representations of word meaning [77–79] and compositional semantics [80]. Of particular relevance to the present work, recurrent neural networks—referred to as the “temporal analogue” of deep neural networks [81]—have been successfully used to model sequential dependencies in language. By applying a variant of Hessian-free optimization to recurrent neural networks, Sutskever et al. [82] surpassed the previous state-of-the-art performance in character-level language modeling. Similarly, Mikolov et al. [80] achieved new state-of-the-art performance on the Microsoft Research Sentence Completion challenge with a weighted combination of an order-sensitive neural network language model and a recurrent neural network language model.

The improvements in performance achieved by deep learning methods over the past decade and the variety of tasks on which these improvements have been realized are such that deep learning has been referred to as a “breakthrough” in machine learning within academia and the popular press [74]. However, reducing the computational complexity of training deep networks remains an active area of research, and deep networks have not been compared with human performance on “semantic” behavioral tasks (e.g., semantic priming and replicating human semantic judgments) as thoroughly as have most of the SSMs described previously in this section. Furthermore, although deep learning methods have several properties that are appealing from a cognitive perspective [73], researchers in machine learning are typically more concerned with a method's performance and mathematical properties than its cognitive plausibility. Given the similarity in the ultimate goals of both approaches—the development of unsupervised and semisupervised methods to compute vector representations of word meaning—cognitive scientists and machine learning researchers alike may benefit from increased familiarity with the most popular methods in each other's fields. This is particularly true given that both fields often settle on similar research questions, for example, how best to integrate distributional lexical statistics with information from other modalities. Similar to findings in cognitive science demonstrating that better fits to human data are achieved when a distributed model is trained simultaneously (rather than separately) on textual data and data derived from perceptual descriptions [27], performance with deep networks is improved when learning features for one modality (e.g., video) with features corresponding to a second modality (e.g., audio) simultaneously rather than in isolation [83].

One of the earliest large-scale SSMs to integrate sequential information into a lexical representation was the Hyperspace Analogue to Language model (HAL; [3]), and it has been proposed that HAL produces lexical organization akin to what a large-scale recurrent network would when trained on language corpora [84]. HAL essentially tabulates a word-by-word cooccurrence matrix in which cell entries are inversely weighted by distance within a moving window (typically 5–10 words) slid across a text corpus. A word's final lexical representation is the concatenation of its row (words preceding target) and column (words succeeding target) vectors from the matrix, normalized by length to reduce the effect of marginal frequency. Typically, columns with the lowest variance are removed prior to concatenation to reduce dimensionality. HAL has inspired several related models for tabulating context word distances (e.g., [50, 85, 86]), and this general class of model has seen considerable success at mimicking human data from sources as diverse as deep dyslexia [87], lexical decision times [88], semantic categorization [15, 89], and information flow [90].

Topic models (e.g., [18]) have seen a recent surge of popularity in modeling the semantic topics from which linguistic contexts could be generated. Topic models have been very successful at explaining high-level semantic phenomena such as the structure of word association norms, but they have also previously been integrated with hidden-Markov models to simultaneously learn sequential structure [48, 91]. These models either independently infer a word's meaning and its syntactic category [91] or infer a hierarchical coupling of probability distributions for a word's topic context dependent on its sequential state. Although promising formal approaches, neither model has yet been applied to model behavioral data.

An alternative approach to encoding temporal information in vector representations is to use vector binding based on high-dimensional random representations (for a review, see [92]). Two random binding models that have been successfully applied to language corpora are the bound encoding of the aggregate language environment model (BEAGLE; [19]) and the random permutation model (RPM; [17]). BEAGLE and RPM can both be loosely thought of as noisy *n*-gram models. Each uses a dedicated function to associate two contiguous words in a corpus but may recursively apply the same function to create vectors representing multiple chunks. For example, in the short phrase “Mary loves John,” an associative operator can be used to create a new vector that represents the *n*-grams Mary-loves and (Mary-loves)-John. The continuous binding of higher-order *n*-grams from a single operator in this fashion is remarkably simple but produces very sophisticated vector representations that contain word transition information. In addition, the associative operations themselves may be inverted to retrieve from memory previously stored associations. Hence, given the probe Mary____John, the operation can be inverted to retrieve plausible words that fit this temporal context from the training corpus that are stored in a distributed fashion in the vector. The applications of BEAGLE and RPM to natural language processing tasks have been studied extensively elsewhere. The focus of this current set of experiments is to study their respective association operators in depth.

Rather than beginning with a word-by-document matrix, BEAGLE and RPM each maintain a static randomly generated* signal* vector for each word in the lexicon. A word's signal vector is intended to represent the mental representation elicited by its invariant physical properties such as orthography and phonology. In both models, this signal structure is assumed to be randomly distributed across words in the environment, but vectors with realistic physical structure are also now possible and seem to enhance model predictions [93].

BEAGLE and RPM also maintain dynamic* memory* vectors for each word. A word's memory representation is updated each time it is experienced in a semantic context as the sum of the signal vectors for the other words in the context. By this process, a word's context is a mixture of the other words that surround it (rather than a frequency tabulation of a document cooccurrence), and words that appear in similar semantic context will come to have similar memory representations as they have had many of the same random signal vectors summed into their memory representations. Thus, the dimensional reduction step in these models is implicitly achieved by superposition of signal vectors and seems to accomplish the same inductive results as those attained by dimensional reduction algorithms such as in LSA, but without the heavy computational requirements [49, 94]. Because they do not require either the overhead of a large word-by-document matrix or computationally intensive matrix decomposition techniques, both BEAGLE and RPM are significantly more scalable than traditional SSMs. For example, encoding with circular convolution in BEAGLE can be accomplished in *O*(*k*log⁡*k*) time, where *k* is a constant representing the number of dimensions in the reduced representation [95], and in *O*(*k*) time with random permutation. By contrast, the complexity of LSA is *O*(*z* + *k*), where *z* is the number of nonzero entries in the matrix and *k* is the number of dimensions in the reduced representation [96]. Critically, *z* increases roughly exponentially with the number of documents [97]. Scalable and incremental random vector accumulation has been shown to be successful on a range of experimental tasks without being particularly sensitive to the choice of parameters such as dimensionality [21, 94, 98, 99].

To represent statistical information about the temporal order in which words are used, BEAGLE and RPM bind together *n*-gram chunks of signal vectors into composite* order* vectors that are added to the memory vectors during training. Integrating information about a word's sequential context (*where* words tend to appear around a target) in BEAGLE has produced greater fits to human semantic data than only encoding a word's discourse context (*what* words tend to appear around a target; [19, 49]). Similarly, Sahlgren et al. [17] report superior performance when incorporating temporal information about word order. Hence, in both models, a word's representation becomes a pattern of elements that reflects both its history of cooccurrence with and position relative to, other words in linguistic experience. Although BEAGLE and RPM differ in respects such as vector dimensionality and chunk size, arguably the most important difference between them is the binding operation used to create order vectors.

BEAGLE uses the operation of circular convolution to bind together signal vectors into a* holographic reduced representation* (HRR; [95, 100]) of *n*-gram chunks that contain each target word. Convolution is a binary operation (denoted by ⊛) performed on two vectors such that every element ${\vec{z}}_{i}$ of $\vec{z}=(\vec{x}⊛\vec{y})$ is given by

(1) $$\begin{array}{c} {\vec{z}}_{i}=\overset{D-1}{\underset{j-0}{\sum }}{\vec{x}}_{j\text{mo}{\text{d}}_{D}}\cdot {\vec{y}}_{(i-j)\text{mo}{\text{d}}_{D}}, \end{array}$$

where *D* is the dimensionality of $\vec{x}$ and $\vec{y}$. Circular convolution can be seen as a modulo-*n* variation of the tensor product of two vectors $\vec{x}$ and $\vec{y}$ such that $\vec{z}$ is of the same dimensionality as $\vec{x}$ and $\vec{y}$. Furthermore, although $\vec{z}$ is dissimilar from both $\vec{x}$ and $\vec{y}$ by any distance metric, approximations of $\vec{x}$ and $\vec{y}$ can be retrieved via the inverse operation of correlation (not related to Pearson's *r*); for example, $\vec{y}\approx \vec{x}\;\#\;\vec{z}$. Hence, not only can BEAGLE encode temporal information together with contextual information in a single memory representation, but also it can invert the temporal encoding operation to retrieve grammatical information directly from a word's memory representation without the need to store grammatical rules (see [19]). Convolution-based encoding and decoding have many precedents in memory modeling (e.g., [101–106]) and have played a key role in models of many other cognitive phenomena as well (e.g., audition [107]; object perception [108]; perceptual-motor skills [109]; reasoning [110]).

In contrast to convolution, RPM employs the unary operation of* random permutation* (RP; [17]) to encode temporal information about a word. RPs are functions that map input vectors to output vectors such that the outputs are simply randomly shuffled versions of the inputs:

(2) $$\begin{array}{c} \Pi :\vec{x}⟶{\vec{x}}^{*}, \end{array}$$

such that the expected correlation between $\vec{x}$ and ${\vec{x}}^{*}$ is zero. Just as $\left(\vec{x}⊛\vec{y}\right)$ produces a vector that differs from $\vec{x}$ and $\vec{y}$ but from which approximations of $\vec{x}$ and $\vec{y}$ can be retrieved, the sum of two RPs of $\vec{x}$ and $\vec{y}$, $\left(\overset{⃑}{z}=\Pi \vec{x}+\Pi^{2}\vec{y}\right)$, where Π^2^ *y* is defined as Π(Π*y*), produces a vector $\overset{⃑}{z}$ dissimilar from $\vec{x}$ and $\vec{y}$ but from which approximations of the original $\vec{x}$ and $\vec{y}$ can be retrieved via $\Pi^{-1}\vec{z}$ and $\Pi^{-2}\vec{z}$, respectively.

Both convolution and random permutation offer efficient storage properties, compressing order information into a single composite vector representation, and both encoding operations are reversible. However, RPs are much more computationally efficient to compute. In language applications of BEAGLE, the computationally expensive convolution operation is what limits the size of a text corpus that the model can encode. As several studies [16, 17, 52] have demonstrated, scaling a semantic model to more data produces much better fits to human semantic data. Hence, both order information and magnitude of linguistic input have been demonstrated to be important factors in human semantic learning. If RPs prove comparable to convolution in terms of storage capacity, performance on semantic evaluation metrics, and cognitive plausibility, the scalability of RPs to large datasets may afford the construction of vector spaces that better approximate human semantic structure while preserving many of the characteristics that have made convolution attractive as a means of encoding order information.

For scaling to large corpora, the implementation of RPs in semantic space models is more efficient than that of circular convolution. This is partly due to the higher computational complexity of convolution with respect to vector dimensionality. Encoding *k*-dimensional bindings with circular convolution can be accomplished in *O*(*k*log⁡*k*) time [95] by means of the fast Fourier transform (FFT). The algorithm to bind two vectors *a* and *b* in *O*(*k*log⁡*k*) time involves calculating discrete Fourier transforms of *a* and *b*, multiplying them pointwise to yield a new vector *c*, and calculating the inverse discrete Fourier transform of *c*. In the BEAGLE model, storing a single bigram (e.g., updating the memory vector of “fox” upon observing “red fox”) would require one such *O*(*k*log⁡*k*) binding, as well as the addition of the resulting vector *c* to the memory vector of “fox.”

In contrast, encoding with RPs can be accomplished in *O*(*k*) (i.e., linear) time, as permuting a vector only requires copying the value at every index of the original vector to a different index of another vector of the same dimensionality. For example, the permutation function may state that the first cell in the original vector should be copied to the 1040th cell of the new vector that the next should be copied to the 239th cell of the new vector, and so on. Thus, this process yields a new vector that contains a shuffled version of the original vector, in a number of steps that scales linearly with vector dimensionality. To update the memory vector of “fox” upon observing “red fox,” RPM would need to apply this process to the environmental vector of “red,” yielding a new shuffled version that would then be added to the memory vector of “fox.”

In addition to the complexity difference, the calculations involved in the FFT implementation of convolution require more time to execute on each vector element than the copy operations involved in random permutation. Combining these two factors means that circular convolution is considerably less efficient than random permutation in practice. In informal empirical comparisons using the FFT routines in a popular open-source mathematics library (http://math.net/), we found circular convolution to be over 70 times slower than random permutation at a vector dimensionality of 2,048. Due to convolution's greater computational complexity, the gap widened even further as dimensionality increased. These factors made it impossible to perform our simulations with BEAGLE on the large corpus.

We conducted four experiments intended to compare convolution and RP as means of encoding word order information with respect to performance and scalability. In Experiment 1, we conducted an empirical comparison of the storage capacity and the probability of correct decoding under each method. In Experiment 2, we compared RP with convolution in the context of a simple vector accumulation model equivalent to BEAGLE's “order space” on a battery of semantic evaluation tasks when trained on a Wikipedia corpus. The model was trained on both the full corpus and a smaller random subset; results improved markedly when RP is allowed to scale up to the full Wikipedia corpus, which proved to be intractable for the convolution-based HRR model. In Experiment 3, we specifically compared BEAGLE to RPM, which differs from BEAGLE in several important ways other than its binding operation, to assess whether using RP in the context of RPM improves performance further. Finally, Experiment 4 demonstrates that similar results can be achieved with random permutations when the constraint that every unit of the input must be mapped to a unique output node is removed. We conclude that RP is a promising and scalable alternative to circular convolution in the context of vector space models of semantic memory and has properties of interest to computational modelers and researchers interested in memory processes more generally.

## 3. Experiment  1: Associative Capacity of HRR and RP

Plate [95] made a compelling case for the use of circular convolution in HRRs of associative memory, demonstrating its utility in constructing distributed representations with high storage capacity and high probability of correct retrieval. However, the storage capacity and probability of correct retrieval with RPs have not been closely investigated. This experiment compared the probability of correct retrieval of RPs with that of circular convolution and explored the how the memory capacity of RPs varies with respect to dimensionality, number of associations stored, and the nature of the input representation.

### 3.1. Method

As a test of the capacity of convolution-based associative memories, Plate [95, Appendix  D] describes a simple paired-associative memory task in which a retrieval algorithm must select the vector ${\vec{x}}_{i}$ that is bound to its associate ${\vec{y}}_{i}$ out of a set *E* of *m* possible random vectors. The retrieval algorithm is provided with a memory vector of the form:

(3) $$\begin{array}{c} \vec{M}=\overset{k}{\underset{i=1}{\sum }}\left({\vec{x}}_{i}⊛{\vec{y}}_{i}\right) \end{array}$$

that stores a total of *k* vectors. All vectors are of dimensionality *D*, and each of ${\vec{x}}_{i}$ and ${\vec{y}}_{i}$ is a normally distributed random vector, i.i.d. with elements sampled from $N\left(0,1/\sqrt{D}\right)$. The retrieval algorithm is provided with the memory vector, $\vec{M}$, and the probe ${\vec{y}}_{i}$, and works by first calculating $\vec{a}=\left({\vec{y}}_{i}\#\vec{M}\right)$, where # is the* correlation operator* described in detail in Plate [95, pp. 94–97], an approximate inverse of convolution. The algorithm then retrieves the vector in the “clean-up memory” set *E* that is the most similar to $\vec{a}$. This is accomplished by calculating the cosine between $\vec{a}$ and each vector in the set *E* and retrieving the vector from *E* for which the cosine is highest. If this vector is not equal to ${\vec{x}}_{i}$, this counts as a retrieval error. We replicated Plate's method to empirically derive retrieval accuracies for a variety of choices of *k* and *D*, keeping *m* fixed at 1,000.

Sahlgren et al. [17] bind signal vectors to positions by means of successive self-composition of a permutation function Π and construct memory vectors by superposing the results. In contrast to circular convolution, which requires normally distributed random vectors, random permutations support a variety of possible inputs. Sahlgren et al. employ* random ternary vectors*, so-called because elements take on one of three possible values (+1, 0, or −1). These are sparse vectors or “spatter codes” [111, 112] whose elements are all zero, with the exception of a few randomly placed positive and negative values (e.g., two +1s and two −1s). In this experiment, we tested the storage capacity of an RP-based associative memory first with normally distributed random vectors (*Gaussian vectors*) to allow a proper comparison to convolution and second with random ternary vectors (*sparse vectors*) with a varying number of positive and negative values in the input.

As for the choice of the permutation function itself, any function that maps each element of the input onto a different element of the output will do; vector rotation (i.e., mapping element *i* of the input to element *i* + 1 of the output, with the exception of the final element of the input, which is mapped to the first element of the output) may be used for the sake of efficiency [17]. Using the notation of function exponentiation employed in our previous work [17, 113], $\Pi^{n}\vec{x}$ refers to Π composed with itself *n* times; $\Pi^{2}\vec{x}=\Pi \left(\Pi \vec{x}\right)$, $\Pi^{3}\vec{x}=\Pi^{2}\left(\Pi \vec{x}\right)$, and so forth. The notion of a memory vector of paired associations can then be recast in RP terms as follows:

(4) M→=Πy→1+Π2x→1+Π3y→2+Π4x→2+Π5y→3+Π6x→3+⋯,

where the task again is to retrieve some ${\overset{⃑}{y}}_{i}$'s associate ${\vec{x}}_{i}$ when presented only with ${\overset{⃑}{y}}_{i}$ and $\vec{M}$. A retrieval algorithm for accomplishing this can be described as follows: given a probe vector ${\overset{⃑}{y}}_{i}$, the algorithm applies the inverse of the initial permutation to memory vector $\vec{M}$, yielding $\Pi^{-1}\vec{M}$. Next, the cosine between $\Pi^{-1}\vec{M}$ and the probe vector ${\overset{⃑}{y}}_{i}$ is calculated, yielding a value that represents the similarity between ${\overset{⃑}{y}}_{i}$ and $\Pi^{-1}\vec{M}$. The previous steps are then iterated: the algorithm calculates the cosine between ${\overset{⃑}{y}}_{i}$ and $\Pi^{-2}\vec{M}$, between ${\overset{⃑}{y}}_{i}$ and $\Pi^{-3}\vec{M}$, and so forth, until this similarity value exceeds some high threshold; this indicates that the algorithm has “found” ${\overset{⃑}{y}}_{i}$ in the memory. At that point, $\vec{M}$ is permuted one more time, yielding ${\vec{x}}^{\prime }$, a noisy approximation of ${\overset{⃑}{y}}_{i}$'s associate ${\vec{x}}_{i}$. This approximation ${\vec{x}}^{\prime }$ can then be compared with clean-up memory to retrieve the original associate ${\vec{x}}_{i}$.

Alternatively, rather than selecting a threshold, *t*_RP_ may be permuted some finite number of times, having its cosine similarity to *y*_*i*_ stored after each permutation. In Plate's [95, p. 252] demonstration of the capacity of convolution-based associative memories, the maximal number of pairs stored in a single memory vector was 14; we likewise restrict the maximal number of pairs in a single memory vector to 14 (i.e., 28 vectors total). Let *n* be the inverse permutation *n* for which cosine(Π^−*n*^ *t*_RP_, *y*_*i*_) was the highest. We can permute one more time to retrieve Π^−*n*−1^ *t*_RP_, that is, our noisy approximation *x*′. This method is appropriate if we always want our algorithm to return an answer (rather than, say, timing out before the threshold is exceeded) and is the method we used for this experiment.

The final clean-up memory step is identical to that used by Plate [95]: we calculate the cosine between *x*′ and each vector in the clean-up memory *E* and retrieve the vector in *E* for which this cosine is highest. As when evaluating convolution, we keep *m* (the number of vectors in *E*) fixed at 1,000 while varying the number of stored vectors *k* and the dimensionality *D*.

### 3.2. Results and Discussion

**Figure 1:**
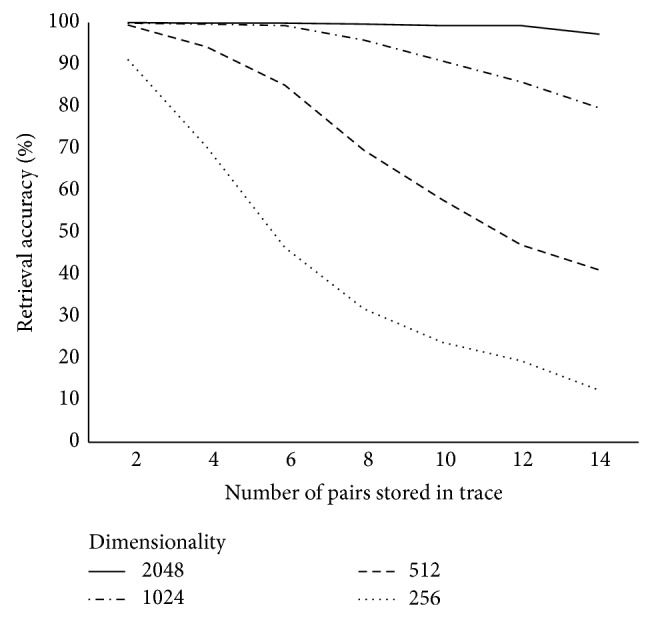
Retrieval accuracies for convolution-based associative memories with Gaussian vectors.

**Figure 2:**
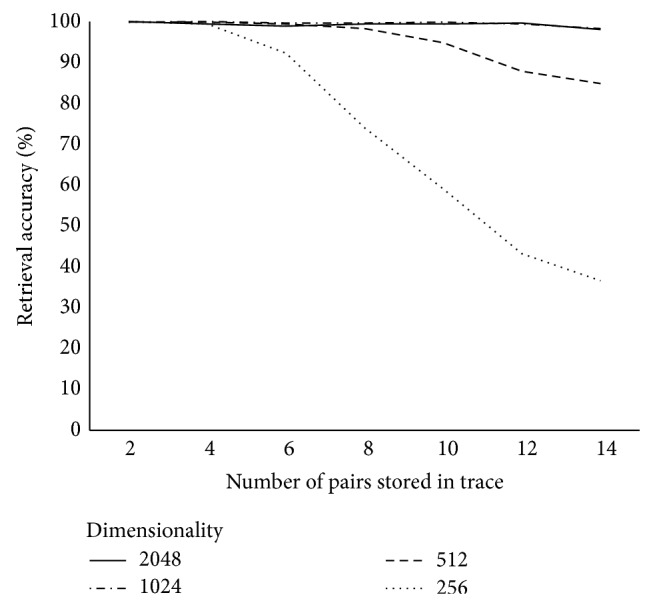
Retrieval accuracies for RP-based associative memories with Gaussian vectors.

Five hundred pairs of normally distributed random vectors were sampled with replacement from a pool of 1,000 and the proportion of correct retrievals was computed. All 1,000 vectors in the pool were potential candidates for retrieval; an accuracy level of 0.1% would represent chance performance.  Figure 1 reports retrieval accuracies for the convolution-based algorithm, while Figure 2 reports retrieval accuracies for the RP formulation of the task. A 2 (algorithm: convolution versus random permutations) × 4 (dimensionality: 256, 512, 1024, 2048) ANOVA with number of successful retrievals as the dependent variable revealed a main effect of algorithm, *F*(1,48) = 11.85, *P* = 0.001, with more successful retrievals when using random permutations (*M* = 457, SD = 86) than when using circular convolution (*M* = 381, SD = 145). There was also a main effect of dimensionality, *F*(3,48) = 18.9, *P* < 0.001. The interaction was not significant, *F*(3,48) = 2.60, *P* = 0.06. Post hoc Tukey's HSD tests showed a significantly lower number of successful retrievals with vectors of dimensionality 256 than with any other vector dimensionality at an alpha of 0.05. All other comparisons were not significant.

**Figure 3:**
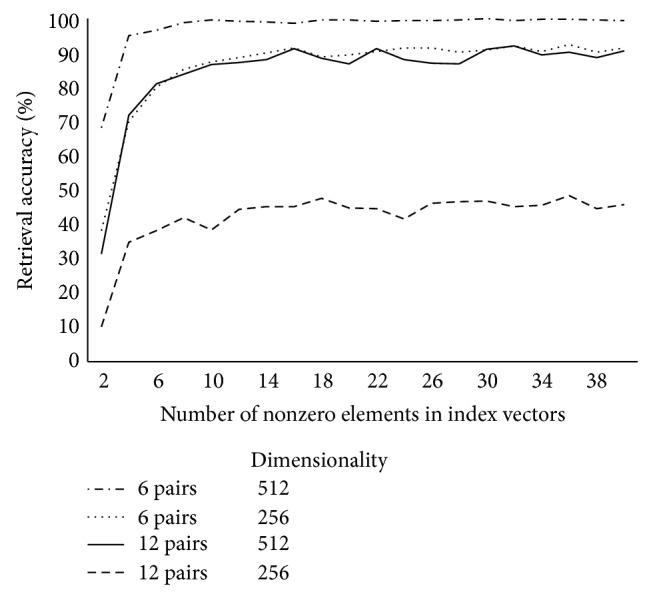
Retrieval accuracies for RP-based associative memories with sparse vectors. The first number reported in the legend (6 or 12) refers to the number of pairs stored in a single memory vector, while the other (256 or 512) refers to the vector dimensionality.

**Figure 4:**
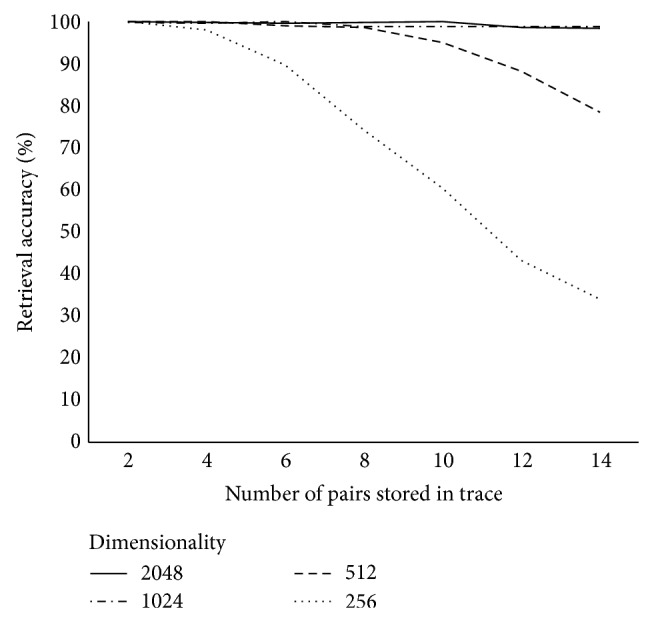
Retrieval accuracies for RP-based associative memories with sparse vectors.

Figure 3 reports retrieval accuracies for RPs when sparse (ternary) vectors consisting of zeroes and an equal number of randomly placed −1 and +1s were used instead of normally distributed random vectors. This change had no impact on performance. A 2 (vector type: normally distributed versus sparse) × 4 (dimensionality: 256, 512, 1024, 2048) ANOVA was conducted with number of successful retrievals as the dependent variable. The main effect of vector type was not significant, *F*(1,48) = 0.011, *P* = 0.92, revealing a nearly identical number of successful retrievals when using normally distributed vectors (*M* = 457, SD = 86) as opposed to sparse vectors (*M* = 455, SD = 88). There was a main effect of dimensionality, *F*(3,48) = 13.0, *P* < 0.001, and the interaction was not significant, *F*(3,48) = 0.004, *P* = 1. As before, post hoc Tukey's HSD tests showed a significantly lower number of successful retrievals with vectors of dimensionality 256 than with any other vector dimensionality at an alpha of 0.05, and all other comparisons were not significant. Figure 3 plots retrieval accuracies against the number of nonzero elements in the sparse vectors, demonstrating that retrieval accuracies level off after the sparse input vectors are populated with more than a handful of nonzero elements.  Figure 4 parallels Figures 1 and 2, reporting retrieval accuracies for RPs at a variety of dimensionalities when sparse vectors consisting of twenty nonzero elements were employed.

Circular convolution has an impressive storage capacity and excellent probability of correct retrieval at high dimensionalities, and our results were comparable to those reported by Plate [95, p. 252] in his test of convolution-based associative memories. However, RPs seem to share these desirable properties as well. In fact, the storage capacity of RPs seems to drop off more slowly than does the storage capacity of convolution as dimensionality is reduced. The high performance of RPs is particularly interesting given that RPs are computationally efficient with respect to basic encoding and decoding operations.

An important caveat to these promising properties of RPs is that permutation is a unary operation, while convolution is binary. While the same convolution operation can be used to unambiguously bind multiple pairs in the convolution-based memory vector *t*, the particular permutation function employed essentially indexed the order in which an item was added to the RP-based memory vector *t*_RP_. This is why the algorithm used to retrieve paired associates from *t*_RP_ necessarily took on the character of a sequential search whereby vectors stored in memory were repeatedly permuted (up to some finite number of times corresponding to the maximum number of paired associates presumed to be stored in memory) and compared to memory. We do not mean to imply that this is a plausible model of how humans store and retrieve paired associates. Experiment 1 was intended merely as a comparison of the theoretical storage capacity of vectors in an RP-based vector space architecture to those in a convolution-based one, and the results do not imply that this is necessarily a superior method of storing paired associates. In RPM, RPs are not used to store paired associates but rather to differentiate cue words occurring in different locations relative to the word being encoded. As a result, pairs of associates cannot be unambiguously extracted from the ultimate space. For example, suppose the word “bird” was commonly followed by the words “has wings” and in an equal number of contexts by the words “eats worms.” While the vector space built up by RPM preserves the information that “bird” tends to be immediately followed by “has” and “eats” rather than “worms” or “wings,” it does not preserve the bindings; “bird eats worms” and “bird eats wings” would be rated as equally plausible by the model. Therefore, one might expect RPM to achieve poorer fits to human data (e.g., synonymy tests, Spearman rank correlations with human semantic similarity judgments) than a convolution-based model. In order to move from the paired-associates problem of Experiment 1 to a real language task, we next evaluate how a simple vector accumulation model akin to Jones and Mewhort's [19] encoding of order-only information in BEAGLE would perform on a set of semantic tasks if RPs were used in place of circular convolution.

## 4. Experiment  2: HRR versus RP on Linguistic Corpora

In this study, we replaced the circular convolution component of BEAGLE with RPs so that we could quantify the impact that the choice of operation alone had on fits to human-derived judgments of semantic similarity. Due to the computational efficiency of RPs, we were able to scale them to a larger version of the same corpus and simultaneously explore the effect of scalability and the method by which order information was encoded.

### 4.1. Method

Order information was trained using both the BEAGLE model and a modified implementation of BEAGLE in which the circular convolution operation was replaced with RPs as they are described in Sahlgren et al. [17]. A brief example will illustrate how this replacement changes the algorithm. Recall that in BEAGLE, each word *w* is assigned a static “environmental” signal vector *e*_*w*_ as well as a dynamic memory vector *m*_*w*_ that is updated during training. Recall also that the memory vector of a word *w* is updated by adding the sum of the convolutions of all *n*-grams (up to some maximum length *λ*) containing *w*. Upon encountering the phrase “one two three” in a corpus, the memory vector for “one” would normally be updated as follows:

(5) $$\begin{array}{c} m_{\text{one}}=m_{\text{one}}+\left(\Phi ⊛e_{\text{two}}\right)+\left(\Phi ⊛e_{\text{two}}⊛e_{\text{three}}\right), \end{array}$$

where Φ is a placeholder signal vector that represents the word whose representation is being updated. In the modified BEAGLE implementation used in this experiment, the memory vector for “one” would instead be updated as

(6) $$\begin{array}{c} m_{\text{one}}=m_{\text{one}}+\Pi e_{\text{two}}+\Pi^{2}e_{\text{three}}. \end{array}$$

In addition to order information, the complete BEAGLE model also updates memory vectors with context information. Each time a word *w* appears in a document, the sum of all of the environmental signal vectors of words cooccurring with *w* in that document is added to *m*_*w*_. In this experiment, context information is omitted; our concern here is with the comparison between convolution and random permutations with respect to the encoding of order information only. Encoding only order information also allowed us to be certain that any differences observed between convolution and RPs were unaffected by the particular stop list or frequency threshold applied to handle high-frequency words, as BEAGLE uses no stop list or frequency thresholding for the encoding of order information.

The RP-based BEAGLE implementation was trained on a 2.33 GB corpus (418 million tokens) of documents from Wikipedia (from [114]). Training on a corpus this large proved intractable for the slower convolution-based approach. Hence, we also trained both models on a 35 MB, six-million-token subset of this corpus constructed by sampling random 10-sentence documents from the larger corpus without replacement. The vector dimensionality *D* was set to 1,024, the lambda parameter indicating the maximum length of an encoded *n*-gram was set to 5, and the environmental signal vectors were drawn randomly from a normal distribution with *μ* = 0 and $\sigma =1/\sqrt{D}$. Accuracy was evaluated on two synonymy tests: English as a Second Language (ESL) and the Test of English as a Foreign Language (TOEFL) synonymy assessments. Spearman rank correlations to human judgments of the semantic similarity of word pairs were calculated using the similarity judgments obtained from Rubenstein and Goodenough (R: [115]), Miller and Charles (MC: [116]), Resnik (R: [117]), and Finkelstein et al. (F: [118]). A detailed description of these measures can be found in Recchia and Jones [52].

### 4.2. Results and Discussion

Table 1 provides a comparison of each variant of the BEAGLE model. Three points about these results merit special attention. First, there are no significant differences between the performance of convolution and RPs on the small corpus. Both performed nearly identically on F and TOEFL; neither showed any significant correlations with human data on RG, MC, R, nor performed better than chance on ESL. Second, both models performed the best by far on the TOEFL synonymy test, supporting Sahlgren et al.'s [17] claim that order information may indeed be more useful for synonymy tests than tests of semantic relatedness, as paradigmatic rather than syntagmatic information sources are most useful for the former. It is unclear exactly why neither model did particularly well on ESL, as models often achieve scores on it comparable to their scores on TOEFL [52]. Finally, only RPs were able to scale up to the full Wikipedia corpus, and doing so yielded strong benefits for every task.

Note that the absolute performance of these models is irrelevant to the important comparisons. Because we wanted to encode order information only to ensure a fair comparison between convolution and RPs, context information (e.g., information about words cooccurring within the document irrespective of order) was deliberately omitted from the model, despite the fact that the combination of context and order information is known to improve BEAGLE's absolute performance. In addition, to keep the simulation as well-controlled as possible, we did not apply common transformations (e.g., frequency thresholding) known to improve performance on synonymy tests [119]. Finally, despite the fact that our corpus and evaluation tasks differed substantially from those used by Jones and Mewhort [19], we kept all parameters identical to those used in the original BEAGLE model. Although these decisions reduced the overall fit of the model to human data, they allowed us to conduct two key comparisons in a well-controlled fashion: the comparison between the performance of circular convolution and RPs when all other aspects of the model are held constant and the comparison in performance between large and small versions of the same corpus. The performance boost afforded by the larger corpus illustrates that, in terms of fits to human data, model scalability may be a more important factor than the precise method by which order information is integrated into the lexicon. Experiment 3 explores whether the relatively low fits to human data reported in Experiment 2 are improved if circular convolution and random permutations are used within their original models (i.e., the original parametrizations of BEAGLE and RPM, resp.).

## 5. Experiment  3: BEAGLE versus RPM on Linguistic Corpora

In contrast with Experiment 2, our present aim is to compare convolution and RPs within the context of their original models. Therefore, this simulation uses both the complete BEAGLE model (i.e., combining context and order information, rather than order information alone as in Experiment 2) and the complete RPM using the original parameters and implementation details employed by Sahlgren at al. (e.g., sparse signal vectors rather than the normally distributed random vectors used in Experiment 2, window size, and vector dimensionality). In addition, we compare model fits across the Wikipedia corpus from Experiment 2 and the well-known TASA corpus of school reading materials from kindergarten through high school used by Jones and Mewhort [19] and Sahlgren et al. [17].

### 5.1. Method

Besides using RPs in place of circular convolution, the specific implementation of RPM reported by Sahlgren et al. [17] differs from BEAGLE in several ways, which we reimplemented to match their implementation of RPM as closely as possible. A primary difference is the representation of environmental signal vectors: RPM uses sparse ternary vectors, consisting of all 0s, two +1s, and two −1s in place of random Gaussians. Additionally, RPM uses a window size of 2 words on either side for both order and context vectors, contrasting with BEAGLE's window size of 5 for order vectors and entire sentences for context vectors. Sahlgren et al. also report optimal performance when the order-encoding mechanism is restricted to “direction information” (e.g., the application of only two distinct permutations, one for words appearing before the word being encoded, and another for words occurring immediately after, rather than a different permutation for words appearing at every possible distance from the encoded word). Other differences included lexicon size (74,100 words to BEAGLE's 90,000), dimensionality (RPM performs optimally at a dimensionality of approximately 25,000, contrasted with common BEAGLE dimensionalities of 1,024 or 2,048), and the handling of high-frequency words: RPM applies a frequency threshold omitting the 87 most frequent words in the training corpus when adding both order and context information to memory vectors, while BEAGLE applies a standard stop list of 280 function words when adding context information only. We trained our implementation of RPM and the complete BEAGLE model (context + order information) on the Wikipedia subset as well as TASA. Other than the incorporation of context information (as described in Experiment 2) to BEAGLE, all BEAGLE parameters were identical to those used in Experiment 2. Accuracy scores and correlations were likewise calculated on the same battery of tasks used in Experiment 2.

### 5.2. Results and Discussion

Performance of all models on all corpora and evaluation tasks are reported in Table 2. On the small Wikipedia subset, BEAGLE and RPM performed similarly across evaluation tasks, with fits to human data being marginally higher than the versions of BEAGLE trained on order information only from Experiment 2, respectively. As in Experiment 2, only the RP-based model proved capable of scaling up to the full Wikipedia corpus and again achieved much better fits to human data on the large dataset. Consistent with previous research [16], the choice of corpus proved just as critical as the amount of training data, with both models performing significantly better on TASA than on the Wikipedia subset despite a similar quantity of text in both corpora. In some cases, RPM achieved even better fits to human data when trained on TASA than on the full Wikipedia corpus. It is perhaps not surprising that a TASA-trained RPM would result in superior performance on TOEFL, as RPM was designed with RPs in mind from the start and optimized with respect to its performance on TOEFL [17]. However, it is nonetheless intriguing that the version of RPM trained on the full Wikipedia in order space was able to perform well on several tasks that are typically conceived of as tests of semantic relatedness and not tests of synonymy per se. While these results do not approach the high correlations on these tasks achieved by state-of-the-art machine learning methods in computational linguistics, our results provide a rough approximation of the degree to which vector space architectures based on neurally plausible sequential encoding mechanisms can approximate the high-dimensional similarity space encoded in human semantic memory. Our final experiment investigates the degree to which a particularly neurologically plausible approximation of a random permutation function can achieve similar performance to RPs with respect to the evaluation tasks applied in Experiments 2 and 3.

## 6. Experiment  4: Simulating RP with Randomly Connected Nodes

**Figure 5:**
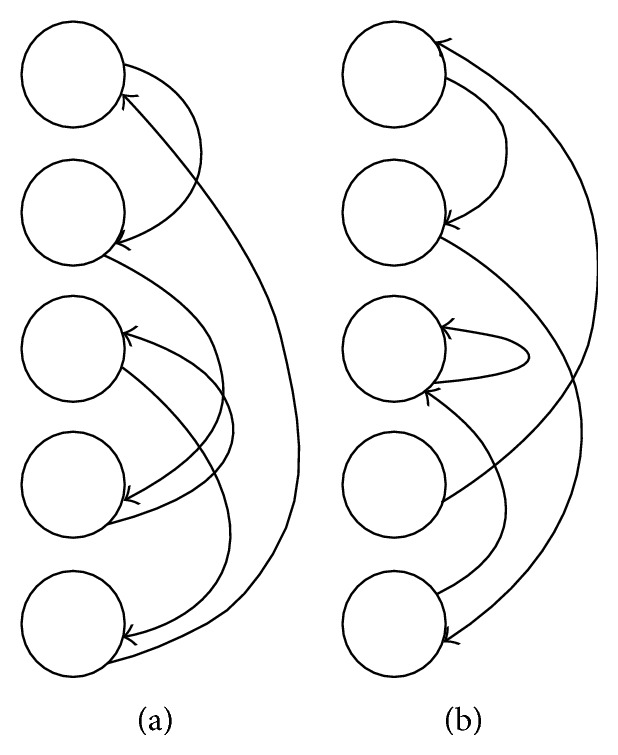
(a) Visual representation of a random permutation function, instantiated by a one-layer recurrent network that maps each node to a unique node on the same layer via copy connections. The network at left would transform an input pattern of 〈0.1,0.2,0.3,0.4,0.5〉 to 〈0.5,0.1,0.4,0.2,0.3〉. (b) A one-layer recurrent network in which each node is mapped to a random node on the same layer, but which lacks the uniqueness constraint of a random permutation function. Multiple inputs feeding into the same node are summed. Thus, the network at right would transform an input pattern of 〈0.1,0.2,0.3,0.4,0.5〉 to 〈0.4,0.1,0.8,0, 0.2〉. At high dimensions, replacing the random permutation function in the vector space model of Sahlgren et al. [17] with an arbitrarily connected network such as this has minimal impact on fits to human semantic similarity judgments (Experiment 4).

Various models of cognitive processes for storing and representing information make use of a random element, including self-organizing feature maps, the Boltzmann machine, randomly connected networks of sigma-pi neurons, and “reservoir computing” approaches such as current popular liquid state models of cerebellar function [92, 120–122]. Random states are employed by such models as a stand-in for unknown or arbitrary components of an underlying structure [92]. Models that make use of randomness in this way should therefore be seen as succeeding in spite of randomness, not because of it. While the use of randomness does not therefore pose a problem for the biological plausibility of mathematical operations proposed to have neural analogues, defending random permutations' status as proper functions whereby each node in the input maps to a* unique* node in the output (Figure 5(a)) would require some biologically plausible mechanism for ensuring that this uniqueness constraint was met. However, this constraint may impose needless restrictions. In this experiment, we investigate random permutation functions with simple* random connections* (RCs) mapping each input node to a random, not necessarily unique node in the same layer (Figure 5(b)). This is equivalent to the use of random sampling with replacement, as opposed to random sampling without replacement. While random sampling without replacement requires some process to be posited by which a single element is not selected twice, random sampling with replacement requires no such constraint. If a model employing random connections (without replacement) can achieve similar fits to human data as the same model employing random permutations, this would eliminate one constraint that might otherwise be a strike against the neural plausibility of the general method.

### 6.1. Method

We replaced the random permutation function in RPM with* random connections*, for example, random mappings between input and output nodes with no uniqueness constraint (e.g., Figure 5(b)). For each node, a unidirectional connection was generated to a random node in the same layer. After one application of the transformation, the value of each node was replaced with the sum of the values of the nodes feeding into it. Nodes with no incoming connections had their values set to zero. The operation can be formally described as a transformation *T* whereby each element of an output vector *y* is updated according to the rule *y*_*k*_ = ∑_*i*_ *x*_*i*_ *w*_*ij*_, in which *w* is a matrix consisting of all zeros except for a randomly placed 1 in every row (with no constraints on the number of nonzero elements present in a single column). As described in Experiment 3, Sahlgren et al. [17] reported the highest fits to human data when encoding “direction information” with a window size of two words on either side of the target (i.e., the application of one permutation to the signal vectors of the two words appearing immediately before the word being encoded and another to the signal vectors two words occurring immediately after it). We approximated this technique with random connections in two different ways. In Simulation 1, we trained by applying the transformation *T* only to the signal vectors of the words appearing immediately* before* the encoded word, applying no transformation to the signal vectors of the words appearing afterward. In Simulation 2, we trained by applying the update rule twice to generate a second transformation *T* ^2^ (cf. Sahlgren et al.'s generation of successive permutation functions Π^2^, Π^3^, etc., via reapplication of the same permutation function); *T* was applied to the signal vectors of the words appearing immediately before the encoded word, while *T* ^2^ was applied to the signal vectors of the words appearing immediately after. All other details of RPM and all evaluation tasks were identical to those used in Experiment 3.

### 6.2. Results and Discussion

Table 3 reports simulation results across the full Wikipedia corpus and TASA.* RC Sim 1* refers to the random connection simulation referred to as Simulation 1 in Methods,* RC Sim 2* to the one referred to as Simulation 2 and RP to the original RPM simulation conducted in Experiment 3. Across tasks, no consistent advantage was observed for the version of RPM using RPs when contrasted with either of the simulations in which RPs were replaced with random connections. These results suggest that the uniqueness constraint is unnecessary for a network of randomly connected nodes to encode order information and that a more biologically plausible approximation of RPs can achieve similar results. However, it is also worth noting that the lack of a one-to-one correspondence between inputs and outputs confers certain disadvantages, such as the lack of an exact inverse. In addition, nodes with no incoming connections will have a value of zero after a single transformation as well as any successive transformations. Thus, in Simulation 2, the number of nodes actively engaged in representing any word is limited to those with at least one incoming connection. For applications of RPs that are restricted to low-dimensional vectors or which require many repetitions of a transformation (e.g., models that require the storage of very long chains of ordered elements without chunking), the removal of the uniqueness constraint may no longer produce comparable results.

## 7. General Discussion

The current study performed a number of controlled comparisons of circular convolution and random permutation (RP), namely, as means of encoding paired associates (Experiment 1) as well as encoding sequential information in word space, both in a single model that differed only in the use of RPs versus convolution (Experiment 2) and in the context of two different models in which each operation had been utilized in previous work (Experiment 3). Finally, a variant of random permutations was explored in which the constraint of a one-to-one mapping between input and output vectors was relaxed (Experiment 4). Experiment 1 showed that RPs are capable of high retrieval accuracy even when many paired associates are stored in a single memory vector, and their storage capacity appears to be better than that of circular convolution for low dimensionalities. Experiments 2 and 3 revealed that both methods achieved approximately equal performance on a battery of semantic tasks when trained on a small corpus, but that RPs were ultimately capable of achieving superior performance due to their higher scalability. Finally, Experiment 4 demonstrated that RPs' uniqueness constraint (e.g., mapping each element of the input to a unique element of the output) is not essential, and a completely random element mapping function can achieve similar fits to human data on the tasks from Experiments 2 and 3.

### 7.1. Cognitive Plausibility in Semantic Space Models

Computational models of human semantic representation vary widely in their goals and the strengths of their assumptions. The strongest claim for a model of semantic memory is that computational units in the algorithm implemented by the model have direct neural correlates, for example, traditional connectionist networks in which the nodes of the network are analogues of individual neurons or populations of neurons. Advances in neurology rendered several of the assumptions of early learning algorithms, such as the reverse connections that would be necessary for backpropagation, largely untenable [123]. Mismatches with human learning abilities, such as the lack of fast mapping, catastrophic interference, and a difficulty with learning systematic rules that can be generalized beyond particular linguistic exemplars, remain problematic for many neural networks as well.

Although we have learned much more about the underpinnings of neural and synaptic function, efforts to make existing vector space models more neurally plausible are few. One notable exception is Gorrell's [55] implementation of an incremental version of LSA that makes use of the Generalized Hebbian Algorithm, a linear feedforward neural network model for unsupervised learning, to derive the decomposition of a mostly unseen word-by-document matrix based on serially presented observations. There are also neurally inspired models of semantic representation that do not attempt to construct a vector space but nonetheless are capable of accounting for some empirical phenomena. These include the work of Murakoshi and Suganuma [124], who present a neural circuit model capable of representing propositions representing general facts (“birds can generally fly”) and implements exceptions (“an ostrich is a bird but cannot fly”) via a model of the spike-timing-dependent plasticity of inhibitory synapses. Cuppini et al. [125] invoke gamma-band synchronization of neural oscillators and a time-dependent Hebbian rule to link lexical representations with collections of multimodal semantic features in a neural network model of semantic memory. On the whole, however, Shepard's [126] observation that connectionist approaches to high-level behavior have assumptions that ignore (and in some cases, contradict) many important properties of neural and synaptic organization continues to ring true today for most models of semantic representation [123].

A model of semantic memory capable of tracing out a path from biological models of synaptic activity to high-level semantic behavior is perhaps the most ambitious goal that a researcher in this field can pursue and the difficulty of bridging these vastly different levels of analysis has attracted few attempts. A more common approach is to develop representational architectures that remain agnostic as to the underlying neural implementation, but which represent information in more abstract ways that are argued to share important high-level properties with human semantic representations. Vector space models are one such approach. Although nodes in a traditional connectionist network for semantic memory often correspond to binary switches indicating the presence or absence of a particular high-level semantic feature, distributed vector representations are more neurally plausible as a basic unit of analysis. However, due to the ease of acquiring large text bases and the difficulty of acquiring reasonable proxies for perceptual and motor representations that contribute to human semantic representations, vector space models of lexical representation are necessarily incomplete. Vector-based representations of semantic properties generated by human participants [127, 128] have been integrated into Bayesian and vector space models to help alleviate this problem [27, 28, 30, 129], but these have their limitations as well, most importantly, the fact that they are mediated by unknown retrieval processes and as such should not be interpreted as providing a direct “snapshot” of a concept's property structure [129]. Thus, although vector space models trained on large text corpora have unavoidable limitations as models of human semantic representation, they at least provide a starting point for a computational approach.

Additionally, close investigation of the properties of vector spaces derived from language data and the ways in which they change in response to different dimensionalities, corpora, and mathematical operations has yielded insights into cooccurrence-based vector spaces that are independent of any particular model (e.g., [16, 119, 130]). Just as Watts and Strogatz's [131] high-level analysis of the fundamental properties of small-world networks has found application in many areas of cognition unanticipated by the original authors, high-level theoretical analyses of the conceptual underpinnings of co-occurrence models in the context of natural language processing (e.g., [132]) strongly influenced their adoption by cognitive modelers such as Andrews et al. [27]. Similarly theoretical analyses of vector space models from a more cognitive perspective [92, 133, 134] have served to clarify the strengths and weaknesses of existing models and may find additional applications as well.

Finally, other works have approached the problem of representing word meaning from a Bayesian perspective, representing word meanings as weightings over a set of probabilistic* topics* [18] within the Latent Dirichlet Allocation (LDA) framework of Blei et al. [135]. Topics are latent variables inferred from observed patterns of word cooccurrence and represent probability distributions over words that tend to cooccur in similar contexts. An individual topic generally turns out to be composed of words that share a common discourse theme (i.e.,* differentiate, calculus, derivative, etc.*) and can be thought of as a more semantically transparent analogue to the reduced dimensions that singular value decomposition yields in LSA. Although the Bayesian approach requires less theoretical commitment to the manner in which such models might be implemented on neurological hardware, it requires a stronger theoretical commitment to the notion that whatever algorithm the brain uses, it is likely to be one that takes advantage of available probabilistic information in an optimal or near-optimal manner. Andrews et al. [27] also work within a Bayesian framework to demonstrate that probabilistic models that take advantage of distributional information (from lexical cooccurrence) as well as experiential (from human-generated semantic feature norms), demonstrating that the joint probability distribution of these two information sources is more predictive of human-based measures of semantic representation than either information source alone or their average.

### 7.2. Cognitive Implications of Holographic Reduced Representation and Random Permutation

Kanerva's [92] review of high-dimensional vector space models and the operations that are used to construct them highlights a number of biologically plausible properties of such models, including their use of lexical representations that are highly distributed, tolerant of noise in the input, and robust to error and component failure. In contrast to the large number of studies focused on LSA and HAL, relatively little work has investigated the properties of lexical representations constructed by means of circular convolution and random permutations, perhaps due in part to the relative youth of BEAGLE and RPM as models of semantic memory. However, there are several cognitively motivated reasons for modelers to be interested in these particular operators as psychologically plausible components of a theory of semantic representation. In traditional connectionist networks (TCNs), an artificial neuron's job is to integrate magnitude information over incoming connections into a scalar value to be forward propagated. While this is one plausible conception of how neurons represent information, it is not the only one. Spike density over a time scale or phase modulations, rather than the magnitude of the electrical signal, may be important factors in information transmission [136]. In holographic neural networks (HNNs; [121]), neurons represent both the magnitude and phase of an incoming pattern with a complex value. HNNs can respond uniquely to different phase patterns, even if they have the same magnitude. Each node contains information about the entire set of stimulus-response pairings, which produces a convolution of stimulus and response signals. The value of the complex node, not the connections, is what is stored in the model. In addition to the many precedents for the use of convolution-based encoding and decoding in memory modeling (e.g., [95, 101–105, 137]) and in models of other cognitive phenomena [107–110], there is evidence that the mathematics of convolution may reflect real operations in the brain, such as the work of Pribram [108], Sutherland [137], and the mathematical framework of neural coding developed by Eliasmith and Anderson [136]. For more recent applications of circular convolution in biologically plausible models of various cognitive phenomena, see Choo and Eliasmith [138], Eliasmith [110], Eliasmith et al. [139], Rasmussen and Eliasmith [140], and Stewart et al. [141].

When formulated as a modulo-*n* tensor product, convolution is computationally expensive, making it difficult to apply to larger models. However, fast Fourier transformations provide a reasonably efficient means of computing convolutions in the frequency domain [95]. The Fourier transform (FT) of the convolution of two functions is equal to the product of their individual FTs, and the product of the FT of one function with the complex conjugate of the FT of the other is equal to the FT of their correlation. Thus, HNNs can be created simply by multiplying the FTs of stimulus and response patterns, calling to mind the brain's tendency to respond to the Fourier components of auditory and spatial stimuli [142], [143, p. 47], and [144].

As previously described, the random permutation model of Sahlgren et al. [17] constitutes an extension to random indexing, which is identical to it in all respects other than the application of random permutations to signal vectors to represent order information. Like LSA, random indexing was not originally conceived of as a cognitive model; Karlgren and Sahlgren [99] emphasize that its impressive performance on evaluation benchmarks suggests that it captures a certain level of functional equivalence, but not necessarily representational equivalence, to the human semantic system. However, the other work has investigated possible points of convergence between human semantic representations and the representations employed by random indexing/RPM in more detail. In particular, the sparse distributed representations employed by RPM and random indexing, which trace their lineage to Kanerva's Sparse Distributed Memory [111], are mathematically compatible with several known properties of neural circuitry. Földiák and Endres [145] provide an excellent review of evidence for sparse codes as a pervasive encoding mechanism within the brain and numerous neural network models. Given recent advances in generative models of lexical representation, it is worth noting that vector space models employing sparse coding should not be interpreted as being antithetical to Bayesian methods. Olshausen and Field [146] demonstrate how measures of sparseness can be interpreted as prior probability distributions and how reconstruction error in clean-up memory can be interpreted in terms of likelihoods. These isomorphisms indicate potential points of convergence between sparse coding models and the Bayesian approach [145].

Random permutations constitute an extension to random indexing that incorporates word order information into what would otherwise be a “bag of words” model, improves performance on semantic tasks [17], and is extremely simple to implement in connectionist terms. A random permutation can simply be thought of as a recurrent one-layer network with randomly placed copy connections that map each input node to a unique node in the same layer. As we will show, this uniqueness constraint is not required; nearly identical results can be achieved with completely random connections (Figure 1). Given that random permutations can be approximated with such simple network structures, it does not seem particularly far-fetched to propose that some analogue of this process may take place within neural tissue.

### 7.3. Conclusion

This paper builds on the work of Kanerva [92] to present the first in-depth analysis of random permutations in the context of models of semantic representation, investigating basic properties such as their storage capacity and computational complexity in a manner analogous to Plate's [95] systematic investigation of holographic reduced representations constructed with circular convolution. In addition, comparing circular convolution and random permutations in the context of semantic memory models affords us a better understanding of two psychologically plausible operations for encoding semantic information that have never been systematically compared.

## Figures and Tables

**Table 1:** Comparisons of variants of BEAGLE differing by binding operation.

Task	Wikipedia subset		Full Wikipedia
	Convolution	Random permutation	Random permutation
ESL	0.20	0.26	0.32
TOEFL	0.46^†^	0.46^†^	0.63^†^
RG	0.07	−0.06	0.32^∗^
MC	0.08	−0.01	0.33^∗^
R	0.06	−0.04	0.35^∗^
F	0.13^∗^	0.12^∗^	0.33^∗^

^∗^Significant correlation, *P* < 0.05, one-tailed.

^†^Accuracy score differs significantly from chance, *P* < 0.05, one-tailed.

Note. For synonymy tests (ESL, TOEFL), values represent the percentage of correct responses.

For all other tasks, values represent Spearman rank correlations between human judgments of semantic similarity and those of the model. Abbreviations for tasks are defined in the main text of the paper.

**Table 2:** Comparison of BEAGLE and RPM by corpus.

Task	Wikipedia subset		TASA		Full Wikipedia
	BEAGLE	RPM	BEAGLE	RPM	RPM
ESL	0.24	0.27	0.30	0.36^†^	0.50^†^
TOEFL	0.47^†^	0.40^†^	0.54^†^	0.77^†^	0.66^†^
RG	0.10	0.10	0.21	0.53^∗^	0.65^∗^
MC	0.09	0.12	0.29	0.52^∗^	0.61^∗^
R	0.09	0.03	0.30	0.56^∗^	0.56^∗^
F	0.23^∗^	0.19^∗^	0.27^∗^	0.33^∗^	0.39^∗^

^∗^Significant correlation, *P* < 0.05, one-tailed.

^†^Accuracy score differs significantly from chance, *P* < 0.05, one-tailed.

Note. For synonymy tests (ESL, TOEFL), values represent the percentage of correct responses.

For all other tasks, values represent Spearman rank correlations between human judgments of semantic similarity and those of the model. Abbreviations for tasks are defined in the main text of the paper.

**Table 3:** Comparison of RPM using random connections (RC) versus random permutations (RP).

Task	Full Wikipedia			TASA		
	RC Sim 1	RC Sim 2	RP	RC Sim 1	RC Sim 2	RP
ESL	0.50^†^	0.48^†^	0.50^†^	0.32	0.32	0.36^†^
TOEFL	0.66^†^	0.66^†^	0.66^†^	0.73^†^	0.74^†^	0.77^†^
RG	0.66^∗^	0.64^∗^	0.65^∗^	0.53^∗^	0.52^∗^	0.53^∗^
MC	0.63^∗^	0.61^∗^	0.61^∗^	0.53^∗^	0.51^∗^	0.52^∗^
R	0.58^∗^	0.56^∗^	0.56^∗^	0.55^∗^	0.54^∗^	0.56^∗^
F	0.39^∗^	0.37^∗^	0.39^∗^	0.32^∗^	0.33^∗^	0.33^∗^

^∗^Significant correlation, *P* < 0.05, one-tailed.

^†^Accuracy score differs significantly from chance, *P* < 0.05, one-tailed.

Note. For synonymy tests (ESL, TOEFL), values represent the percentage of correct responses.

For all other tasks, values represent Spearman rank correlations between human judgments of semantic similarity and those of the model. Abbreviations for tasks are defined in the main text of the paper.
